# Prognostic Nutritional Index Correlates with Liver Function and Prognosis in Chronic Liver Disease Patients

**DOI:** 10.3390/diagnostics14010049

**Published:** 2023-12-25

**Authors:** Masahiro Matsui, Akira Asai, Kosuke Ushiro, Saori Onishi, Tomohiro Nishikawa, Hideko Ohama, Yusuke Tsuchimoto, Soo Ki Kim, Hiroki Nishikawa

**Affiliations:** 1 The Second Department of Internal Medicine, Osaka Medical and Pharmaceutical University, Takatsuki 569-8686, Osaka, Japan; 2 Liver Center, Osaka Medical and Pharmaceutical University, Takatsuki 569-8686, Osaka, Japan; 3 Department of Gastroenterology, Kobe Asahi Hospital, Kobe 653-8501, Hyogo, Japan

**Keywords:** Prognostic Nutritional Index, chronic liver disease, malnutrition, predictor, discriminative ability

## Abstract

The Prognostic Nutritional Index (PNI) is widely recognized as a screening tool for nutrition. We retrospectively examined the impact of PNI in patients with chronic liver disease (CLD, *n* = 319, median age = 71 years, 153 hepatocellular carcinoma (HCC) patients) as an observational study. Factors associated with PNI < 40 were also examined. The PNI correlated well with the albumin–bilirubin (ALBI) score and ALBI grade. The 1-year cumulative overall survival rates in patients with PNI ≥ 40 (*n* = 225) and PNI < 40 (*n* = 94) were 93.2% and 65.5%, respectively (*p* < 0.0001). In patients with (*p* < 0.0001) and without (*p* < 0.0001) HCC, similar tendencies were found. In the multivariate analysis, hemoglobin (*p* = 0.00178), the presence of HCC (*p* = 0.0426), and ALBI score (*p* < 0.0001) were independent factors linked to PNI < 40. Receiver operating characteristic (ROC) curve analysis based on survival for the PNI yielded an area under the ROC curve of 0.79, with sensitivity of 0.80, specificity of 0.70, and an optimal cutoff point of 42.35. In conclusion, PNI can be a predictor of nutritional status in CLD patients. A PNI of <40 can be useful in predicting the prognosis of patients with CLD.

## 1. Introduction

Malnutrition is a condition in which the intake or absorption of nutrients essential for development and the prevention of disease is inadequate [1,2]. Compared to healthy individuals, patients with gastrointestinal diseases often have altered nutrient metabolism [3,4,5,6,7]. In particular, patients with chronic liver diseases (CLDs) frequently become malnourished [8]. More than 844 million people worldwide currently suffer from CLDs, and approximately 2 million CLD patients die annually [8]. The liver is an important organ in which the metabolism and biosynthesis of a multitude of proteins, carbohydrates, and fats take place. Thus, progressive liver disease often results in fat malabsorption, fat-soluble vitamin deficiencies, decreased levels of water-soluble vitamins, and altered micronutrient metabolism [9,10]. In particular, cirrhotic patients have a high rate of protein–energy malnutrition (PEM), sarcopenia, or decreased quality of life [9,10,11,12,13]. Among a cohort of Child–Pugh class A patients, the 1-year mortality rate for those who were malnourished was approximately 20%, but none of the patients who received adequate amounts of nutrition died within 1 year [14]. Malnutrition is known to have a significant impact on patient outcomes [14]. Early detection and treatment of malnutrition may be necessary to improve patient outcomes. It is therefore important to accurately assess the nutritional status of CLD patients.

In this study, we conducted a nutritional evaluation of CLD patients using the Prognostic Nutritional Index (PNI). PNI was first suggested to be a predictor of nutritional status and surgery-related risk by Buzby and colleagues in 1980 and confirmed by Onodera and colleagues in 1984 [15,16]. Since then, it has been further validated, with many recent studies having demonstrated that lower PNI is an independent adverse predictor for surgery-related complications and long-term clinical outcomes in various malignancies, such as gastric cancer, colorectal cancer, and esophageal cancer [17,18,19]. PNI is calculated from only two serum markers: the serum albumin level and lymphocyte count, which can be easily measured in clinical settings. Patients with PNI < 40 are defined as malnourished [16]. The impact of PNI has been previously reported in patients with HCC [20]. However, to our knowledge, little has been reported on the clinical implications of PNI in CLD patients without HCC. Identifying CLD patients at risk for severe malnutrition and providing early nutritional intervention targeting patients at high nutritional risk may improve their prognosis. This clinical research question seems to be worth clarifying the answer. That was the reason for the current analysis.

## 2. Patients and Methods

### 2.1. Patients

This study was a single-center observational study with a retrospective nature. Osaka Medical and Pharmaceutical University (OMPU) Hospital is currently one of the leading CLD high-volume facilities in the country. Data on patients with CLDs have been continuously accumulated in our database. Between May 2020 and May 2023, 319 Japanese CLD patients with calculated PNI admitted to OMPU Hospital were included in the database. Appropriate interventions were made for each condition in all patients. The baseline included variables were as listed in Table 1. Ethics approval was obtained from the Ethics Committee of OMPU Hospital (approval number: 2021-109, approval date: 25 November 2021; details are available on our website). Since this was a retrospective study, direct face-to-face informed consent from the patients with regard to the study was waived. The protocol of the current study strictly adhered to all contents of the 1975 Declaration of Helsinki.

### 2.2. PNI and Our Analysis

PNI can be calculated as follows: 10 × serum albumin (g/L) + 0.005 × lymphocyte count in the peripheral blood (mm^3^) [15,16]. As mentioned earlier, PNI in each patient was calculated. First, we examined the relationship between PNI and the ALBI score [21] for all cases, hepatocellular carcinoma (HCC) cases, and non-HCC cases. Next, PNI and the percentage of patients with PNI < 40 were compared according to the albumin–bilirubin (ALBI) grade or modified ALBI (mALBI) grade [21,22] in all cases, HCC cases, and non-HCC cases. Next, factors associated with PNI < 40 were investigated in uni- and multivariate analyses. Next, we performed a receiver operating characteristic (ROC) curve analysis of independent factors in the multivariate analysis for PNI < 40. Next, the overall survival (OS) ratio was compared, stratified by the baseline PNI. Finally, we performed an ROC curve analysis for PNI based on the prognosis.

### 2.3. Statistical Considerations

For two-group comparisons of continuous parameters, Student’s *t* test or the Mann–Whitney U test was applied as appropriate, and for multiple-group comparisons of continuous parameters, ANOVA or the Kruskal–Wallis test was applied as appropriate. For group comparisons of categorical parameters, the Pearson χ^2^ test was applied. Pearson’s correlation coefficient *r* was used for the correlation coefficient. For a comparison of survival, the Kaplan–Meier method was adopted and tested using the log-rank method. Details for continuous parameters are presented as the median (interquartile range, IQR) unless otherwise noted. Multivariate logistic regression analysis related to PNI < 40 was also performed to identify independent covariates. The significance level was set at 0.05 using JMP ver. 17 (SAS Institute Inc., Cary, NC, USA).

## 3. Results

### 3.1. Patient Baseline Characteristics

The baseline features for all study subjects (*n* = 319, 183 males and 136 females, median (IQR) age = 71 (63.0–78.0) years) are presented in Table 1. In terms of etiologies of CLD patients, alcohol, hepatitis B virus (HBV), hepatitis C virus (HCV), metabolic-dysfunction-associated steatotic liver disease (MASLD), and others were found in 39, 52, 73, 112, and 43 patients, respectively. HCC was seen in 153 cases (48.0%). The median (IQR) body mass index was 22.9 (20.8–25.6) kg/m^2^. The median (IQR) serum albumin level was 3.8 (3.3–4.1) g/dL. The median (IQR) ALBI score was −2.48 (−2.76–2.05). The ALBI grade observed was grade 1 in 122 patients, grade 2 in 176, and grade 3 in 21. The median (IQR) ALBI score was −2.48 (−2.76–2.05). Modified ALBI (mALBI [22]) grade 1 was seen in 122 patients, grade 2a was seen in 79, grade 2b was seen in 97 and grade 3 was seen in 21. The median (IQR) PNI was 44.2 (38.7–48.8). PNI < 40 was seen in 94 cases (29.5%).

### 3.2. The Relevance in PNI and the ALBI Score

**Figure 1:**
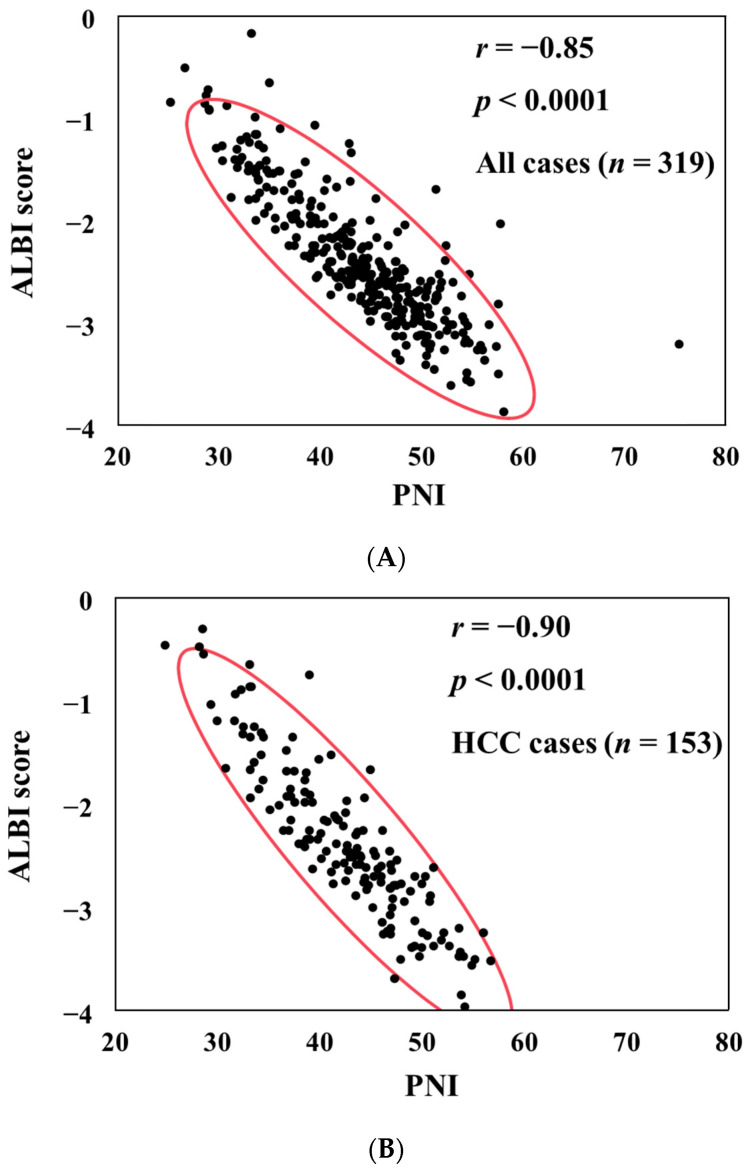

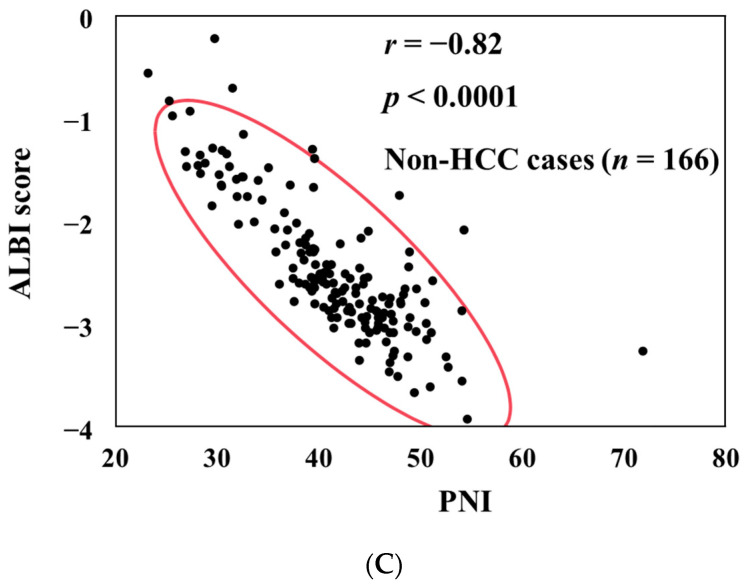
The correlation between PNI and ALBI score for all cases (**A**), HCC cases (**B**), and non-HCC cases (**C**).

PNI had a significant negative correlation with the ALBI score in all study subjects (*r* = −0.85, *p* < 0.0001) (Figure 1A). PNI also had a significant negative correlation with the ALBI score in HCC cases (*r* = −0.90, *p* < 0.0001) (Figure 1B) and in non-HCC cases (*r* = −0.82, *p* < 0.0001) (Figure 1C).

### 3.3. PNI According to ALBI Grade and mALBI Grade in All Cases

**Figure 2:**
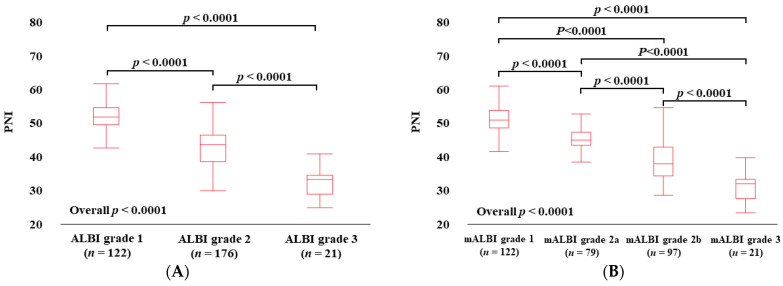

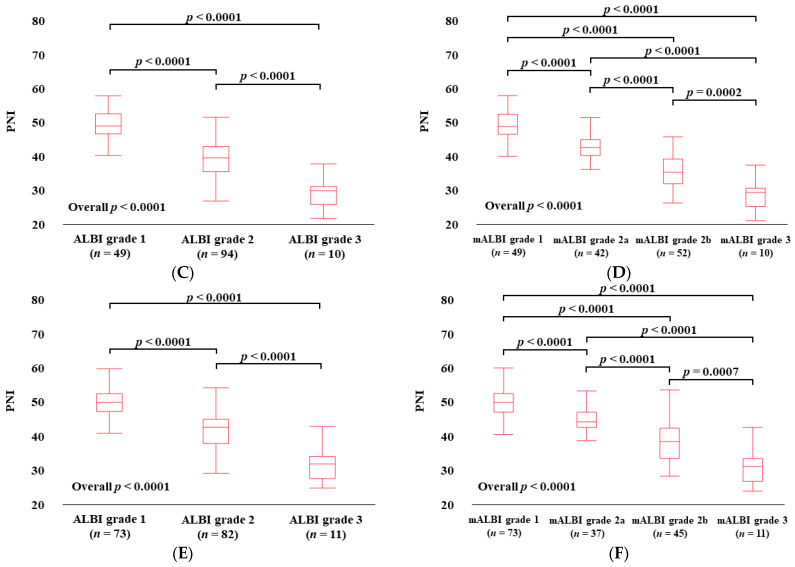
PNI according to ALBI grade (**A**) and modified ALBI grade (**B**) for all cases. PNI according to ALBI grade (**C**) and modified ALBI grade (**D**) for HCC cases. PNI according to ALBI grade (**E**) and modified ALBI grade (**F**) for non-HCC cases.

The median (IQR) PNI values in categories of ALBI grade 1 (*n* = 122), grade 2 (*n* = 176), and grade 3 (*n* = 21) were 49.61 (47.37–52.22) in grade 1, 41.58 (36.62–47.54) in grade 2, and 31.21 (27.06–32.44) in grade 3, (*p* values: ALBI grade 1 vs. 2, *p* < 0.0001; grade 1 vs. 3, *p* < 0.0001; grade 2 vs. 3, *p* < 0.0001; overall *p* < 0.0001) (Figure 2A). The median (IQR) PNI values in categories of mALBI grade 1 (*n* = 122), grade 2a (*n* = 79), grade 2b (*n* = 97), and grade 3 (*n* = 21) were 49.61 (47.37–52.22) in mALBI grade 1, 43.71 (42.26–46.11) in grade 2a, 37.06 (33.40–41.63) in grade 2b, and 31.21 (27.06–32.44) in grade 3 (*p* values: mALBI grade 1 vs. 2a, *p* < 0.0001; grade 1 vs. 2b, *p* < 0.0001; grade 1 vs. 3, *p* < 0.0001; grade 2a vs. 2b, *p* < 0.0001; grade 2a vs. 3, *p* < 0.0001; grade 2b vs. 3, *p* < 0.0001; overall *p* < 0.0001) [22] (Figure 2B).

### 3.4. PNI According to ALBI Grade and mALBI Grade in HCC Cases

The median (IQR) PNI values in categories of ALBI grade 1 (*n* = 49), grade 2 (*n* = 94), and grade 3 (*n* = 10) in HCC cases were 49.67 (47.45–53.17) in ALBI grade 1, 40.38 (36.55–43.87) in grade 2, and 30.89 (26.92–32.20) in grade 3 (*p* values: ALBI grade 1 vs. 2, *p* < 0.0001; grade 1 vs. 3, *p* < 0.0001; grade 2 vs. 3, *p* < 0.0001; overall *p* < 0.0001) (Figure 2C). The median (IQR) PNI values in categories of mALBI grade 1 (*n* = 49), grade 2a (*n* = 42), grade 2b (*n* = 52), and grade 3 (*n* = 10) in HCC cases were 49.67 (47.45–53.17) in mALBI grade 1, 43.69 (41.43–45.92) in grade 2a, 36.73 (33.39–40.52) in grade 2b, and 30.89 (26.92–32.20) in grade 3 (*p* values: mALBI grade 1 vs. 2a, *p* < 0.0001; grade 1 vs. 2b, *p* < 0.0001; grade 1 vs. 3, *p* < 0.0001; grade 2a vs. 2b, *p* < 0.0001; grade 2a vs. 3, *p* < 0.0001; grade 2b vs. 3, *p* = 0.0002; overall *p* < 0.0001) (Figure 2D).

### 3.5. PNI According to ALBI Grade and mALBI Grade after Excluding HCC Cases

The median (IQR) PNI values in categories of ALBI grade 1 (*n* = 73), grade 2 (*n* = 82), and grade 3 (*n* = 11) in non-HCC cases were 49.55 (46.87–51.98) in ALBI grade 1, 42.18 (37.43–44.49) in grade 2, and 31.52 (27.15–33.69) in grade 3 (*p* values: ALBI grade 1 vs. 2, *p* < 0.0001; grade 1 vs. 3, *p* < 0.0001; grade 2 vs. 3, *p* < 0.0001; overall *p* < 0.0001) (Figure 2E). The median (IQR) PNI values in categories of mALBI grade 1 (*n* = 73), grade 2a (*n* = 37), grade 2b (*n* = 45), and grade 3 (*n* = 11) in non-HCC cases were 49.55 (46.87–51.98) in mALBI grade 1, 43.91 (42.46–46.80) in grade 2a, 38.41 (33.73–42.28) in grade 2b, and 31.52 (27.15–33.69) in grade 3 (*p* values: mALBI grade 1 vs. 2a, *p* < 0.0001; grade 1 vs. 2b, *p* < 0.0001; grade 1 vs. 3, *p* < 0.0001; grade 2a vs. 2b, *p* < 0.0001; grade 2a vs. 3, *p* < 0.0001; grade 2b vs. 3, *p* = 0.0007; overall *p* < 0.0001) (Figure 2F).

### 3.6. Patient Frequency with PNI < 40 According to ALBI Grade and mALBI Grade among All Cases

**Figure 3:**
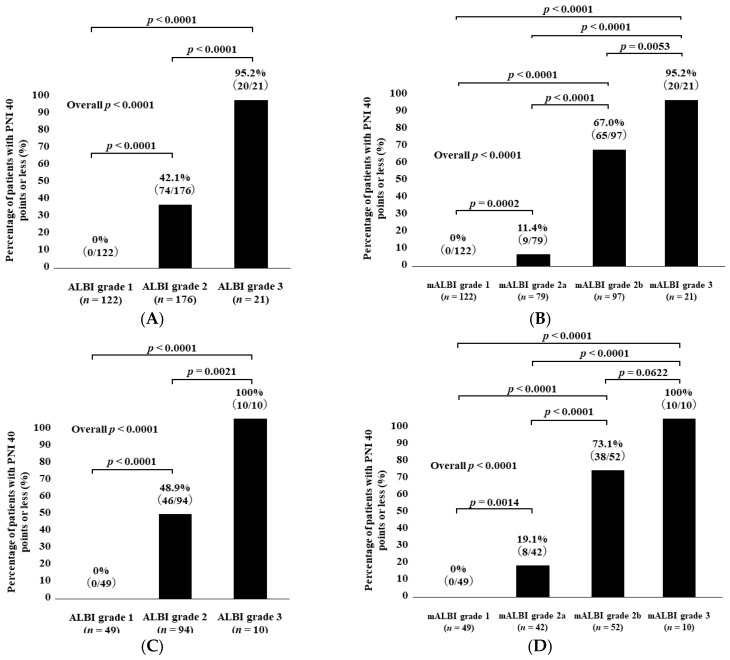

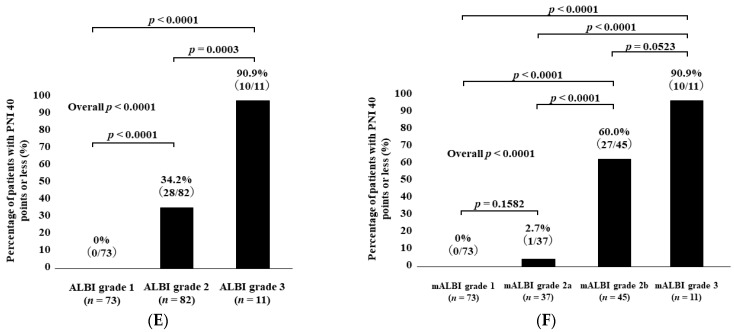
Percentage of patients with PNI < 40 according to ALBI grade (**A**) and modified ALBI grade (**B**) for all cases. Percentage of patients with PNI < 40 according to ALBI grade (**C**) and modified ALBI grade (**D**) for HCC cases. Percentage of patients with PNI < 40 according to ALBI grade (**E**) and modified ALBI grade (**F**) for non-HCC cases.

The percentages of patients with PNI < 40 in categories of ALBI grade 1, grade 2, and grade 3 among all cases were 0% (0/122) in ALBI grade 1, 42.1% (74/176) in grade 2, and 95.2% (20/21) in grade 3 (*p* values: ALBI grade 1 vs. 2, *p* < 0.0001; grade 1 vs. 3, *p* < 0.0001; grade 2 vs. 3, *p* < 0.0001; overall *p* < 0.0001) (Figure 3A).

The percentages of patients with PNI < 40 in categories of mALBI grade 1, grade 2a, grade 2b, and grade 3 among all cases were 0% (0/122) in mALBI grade 1, 11.4% (9/79) in grade 2a, 67.0% (65/97) in grade 2b, and 95.2% (20/21) in grade 3 (*p* values: mALBI grade 1 vs. 2a, *p* = 0.0002; grade 1 vs. 2b, *p* < 0.0001; grade 1 vs. 3, *p* < 0.0001; grade 2a vs. 2b, *p* < 0.0001; grade 2a vs. 3, *p* < 0.0001; grade 2b vs. 3, *p* = 0.0053; overall *p* < 0.0001) (Figure 3B).

### 3.7. Patient Frequency with PNI < 40 According to ALBI Grade and mALBI Grade among HCC Cases

The percentages of patients with PNI < 40 in categories of ALBI grade 1, grade 2, and grade 3 among the HCC cases were 0% (0/49) in ALBI grade 1, 48.9% (46/94) in grade 2, and 100% (10/10) in grade 3 (*p* values: ALBI grade 1 vs. 2, *p* < 0.0001; grade 1 vs. 3, *p* < 0.0001; grade 2 vs. 3, *p* = 0.0021; overall *p* < 0.0001) (Figure 3C).

The percentages of patients with PNI < 40 in categories of mALBI grade 1, grade 2a, grade 2b, and grade 3 among the HCC cases were 0% (0/49) in mALBI grade 1, 19.1% (8/42) in grade 2a, 73.1% (38/52) in grade 2b, and 100% (10/10) in grade 3 (*p* values: mALBI grade 1 vs. 2a, *p* = 0.0014; grade 1 vs. 2b, *p* < 0.0001; grade 1 vs. 3, *p* < 0.0001; grade 2a vs. 2b, *p* < 0.0001; grade 2a vs. 3, *p* < 0.0001; grade 2b vs. 3, *p* = 0.0622; overall *p* < 0.0001) (Figure 3D).

### 3.8. Patient Frequency with PNI < 40 According to ALBI Grade and mALBI Grade after Excluding HCC Cases

The percentages of patients with PNI < 40 in categories of ALBI grade 1, grade 2, and grade 3 among the non-HCC cases were 0% (0/73) in ALBI grade 1, 34.2% (28/82) in grade 2, and 90.9% (10/11) in grade 3 (*p* values: ALBI grade 1 vs. 2, *p* < 0.0001; grade 1 vs. 3, *p* < 0.0001; grade 2 vs. 3, *p* = 0.0003; overall *p* < 0.0001) (Figure 3E).

The percentages of patients with PNI < 40 in categories of mALBI grade 1, grade 2a, grade 2b, and grade 3 among the non-HCC cases were 0% (0/73) in mALBI grade 1, 2.7% (1/37) in grade 2a, 60.0% (27/45) in grade 2b, and 90.9% (10/11) in grade 3 (*p* values: mALBI grade 1 vs. 2a, *p* = 0.1582; grade 1 vs. 2b, *p* < 0.0001; grade 1 vs. 3, *p* < 0.0001; grade 2a vs. 2b, *p* < 0.0001; grade 2a vs. 3, *p* < 0.0001; grade 2b vs. 3, *p* = 0.0523; overall *p* < 0.0001) (Figure 3F).

### 3.9. Uni- and Multivariate Analyses of Covariates Associated with PNI < 40

In the univariate analysis, age (*p* = 0.0107), hemoglobin (Hb, *p* < 0.0001), C-reactive protein (*p* < 0.0001), prothrombin time (*p* < 0.0001), platelet count (*p* = 0.0002), estimated glomerular filtration rate (*p* < 0.0001), presence of HCC (*p* = 0.0072), and ALBI score (*p* < 0.0001) were significant factors related to PNI < 40 (Table 2). Hb (*p* = 0.0017), HCC (*p* = 0.0426), and ALBI score (*p* < 0.0001) were independent factors related to PNI < 40 in the multivariate analysis (Table 3).

### 3.10. ROC Analysis

An ROC analysis of independent continuous covariates in the multivariate analysis for PNI < 40 was performed. The corresponding area under the ROC curve (AUC), sensitivity, specificity, and best reference point for Hb and ALBI score are demonstrated in Table 4. ALBI score involved the highest AUC for PNI < 40 (AUC = 0.96), followed by Hb (AUC = 0.76).

### 3.11. Cumulative OS Rate Stratified by Baseline PNI

**Figure 4:**
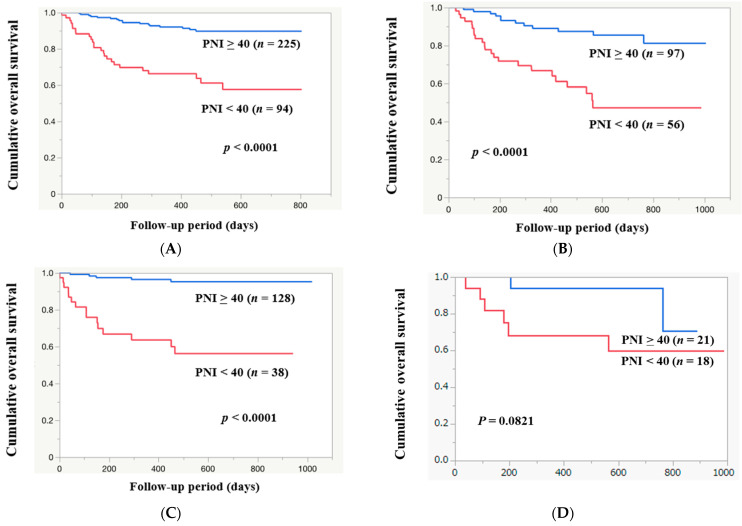

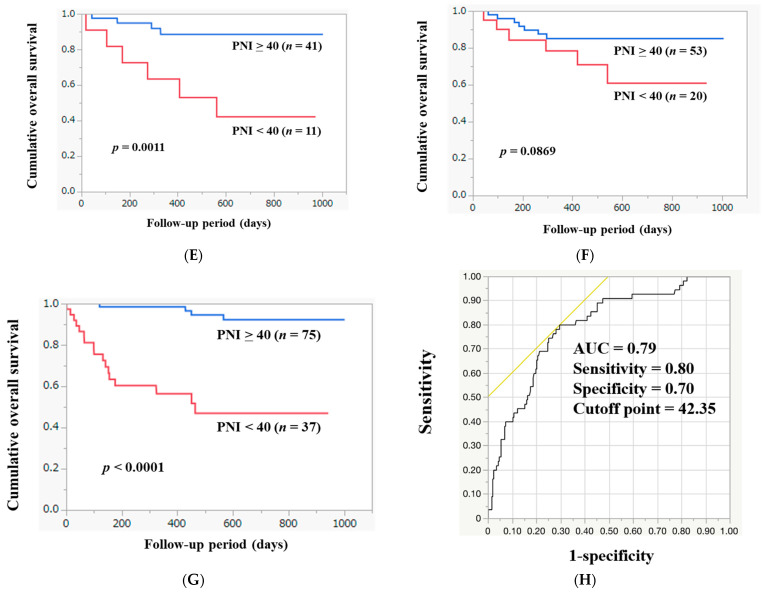
The cumulative OS rates among patients with PNI ≥ 40 and PNI < 40 in all cases (**A**), in HCC cases (**B**), in non-HCC cases (**C**), in alcoholic cases (**D**), in HBV cases (**E**), in HCV cases (**F**), and in MASLD cases (**G**). (**H**) ROC analysis based on the prognosis for PNI.

The median follow-up interval was 524 days. Within the follow-up period, 55 patients (17.2%) died (all were liver-disease-related). The 1-year cumulative OS rate for all study subjects was 84.9%. The 1-year cumulative OS rates among patients with PNI ≥ 40 (*n* = 225) and PNI < 40 (*n* = 94) were 93.2% and 65.5%, respectively (*p* < 0.0001, Figure 4A). Among the HCC cases, the 1-year cumulative OS rate was 77.1%. The 1-year cumulative OS rates among HCC patients with PNI ≥ 40 (*n* = 97) and PNI < 40 (*n* = 56) were 89.0% and 66.7%, respectively (*p* < 0.0001, Figure 4B). After exclusion of 153 HCC cases, the 1-year cumulative OS rate for all cases was 88.0%. The 1-year cumulative OS rates among non-HCC patients with PNI ≥ 40 (*n* = 128) and PNI < 40 (*n* = 38) were 96.4% and 63.6%, respectively (*p* < 0.0001, Figure 4C). Among alcoholic cases (*n* = 39), the 1-year cumulative OS rate was 81.8%. The 1-year cumulative OS rates in alcoholic patients with PNI ≥ 40 (*n* = 21) and PNI < 40 (*n* = 18) were 94.1% and 68.3%, respectively (*p* = 0.0821, Figure 4D). Among HBV cases (*n* = 52), the 1-year cumulative OS rate was 82.9%. The 1-year cumulative OS rates among HBV patients with PNI ≥ 40 (*n* = 41) and PNI < 40 (*n* = 11) were 88.5% and 63.6%, respectively (*p* = 0.0011, Figure 4E). Among HCV cases (*n* = 73), the 1-year cumulative OS rate was 83.3%. The 1-year cumulative OS rates among HCV patients with PNI ≥ 40 (*n* = 53) and PNI < 40 (*n* = 20) were 85.3% and 78.4%, respectively (*p* = 0.0869, Figure 4F). Among MASLD cases (*n* = 112), the 1-year cumulative OS rate was 84.4%. The 1-year cumulative OS rates among MASLD patients with PNI ≥ 40 (*n* = 75) and PNI < 40 (*n* = 37) were 98.5% and 56.4%, respectively (*p* < 0.0001, Figure 4G).

### 3.12. ROC Analysis Based on Death or Survival for PNI

In the ROC analysis based on death or survival for PNI, the AUC was 0.79 (Figure 4H). The sensitivity and specificity were 0.80 and 0.70, respectively, with an optimal cutoff point of 42.35.

## 4. Discussion

In this study, PNI was found to be a predictor of nutritional status in patients with CLD. A PNI value of <40 may be useful in predicting prognosis in patients with CLD. These results are important because PNI may allow for early intervention in nutritional therapy for CLD patients. Early nutritional therapy may contribute to a better prognosis.

Patients with advanced chronic disease are often malnourished and are not able to obtain necessary nutrients through oral intake alone. Inadequate intake and malabsorption in the gastrointestinal tract can lead to poor body composition and biological function [8,23]. Liver disease is no exception, and nutritional status has been recognized as a predictor of prognosis for patients with advanced liver disease [8,24,25,26]. However, nutritional assessment is often neglected in the routine care of CLD patients [27]. Unfortunately, the reality is that nutritional problems in CLD patients are often underestimated, and adequate nutritional assessment is not performed. As a result, nutritional therapy interventions for patients with CLD are often underutilized [27]. Based on this background, it is desirable to assess nutritional status in patients with CLD.

Metabolism 2006 guidelines recommend the use of subjective global assessment (SGA) to identify CLD patients at risk for malnutrition [28]. SGA is a bedside assessment tool to collect information on food intake, weight changes, and gastrointestinal symptoms; it also includes tests for subcutaneous fat loss, muscle weakness, ascites, and edema [8,29]. While SGA is appropriate as a stand-alone nutritional assessment tool, several studies have reported that SGA can underestimate the disease severity and frequency of malnutrition in patients with the early stages of the disease [8,30]. In the dual-energy X-ray absorptiometry method, a living body is irradiated with X-rays of two different energies, and body composition components are determined using the attenuation rate of the irradiated radiation as it passes through the body [28]. Although this provides accurate information, it is not widely used due to its technical complexity and higher cost [8,29]. Currently, there are many challenges with tools used to assess the nutritional status of CLD patients. PNI is a simple, objective indicator of inflammation and nutritional status, calculated from only serum albumin and lymphocyte counts [15,16,31]. In recent studies, PNI has also been significantly correlated with prognosis and nutritional status in a variety of diseases [31]. PNI may be useful in the nutritional assessment of patients with CLD. In the current study, we examined the influence of PNI in patients with CLD. The results of our study presented that PNI correlated strongly with ALBI score (*r* = −0.85) for all study subjects. A similar strong correlation with ALBI score was observed in HCC patients (*r* = −0.90) and non-HCC patients (*r* = −0.82). A comparison of PNI by ALBI grade or mALBI grade in patients with CLD showed a trend of decreasing PNI with worsening liver function. Thus, lower PNI can be associated with liver function. In our data, the percentages of patients with PNI < 40 in groups of ALBI grade 1, grade 2, and grade 3 among all study subjects were 0% (0/122) in ALBI grade 1, 42.1% (74/176) in grade 2, and 95.2% (20/21) in grade 3 (overall *p* < 0.0001). Identical tendencies were observed among HCC and non-HCC patients. PNI thus appears to be robustly associated with hepatic reserve capacity, with or without HCC. In our multivariate analysis, Hb and the presence of HCC, in addition to ALBI score, were significant covariates related to PNI. These results indicate that Hb and the presence of HCC, besides ALBI score, can be reliable markers for malnutrition in CLD patients. First, in terms of Hb, malnutrition is actually a known common cause of anemia, especially in certain clinical conditions such as older age [32,33]. Second, in terms of comorbid malignancies, previous studies have shown that impaired nutritional status frequently occurs in HCC patients. Impaired nutritional status may also be associated with unfavorable outcome in patients with HCC [34,35,36,37]. In terms of survival, the 1-year cumulative OS rates for patients with PNI ≥ 40 and PNI < 40 were 93.2% and 65.5%, respectively (*p* < 0.0001). In HCC cases and non-HCC cases, similar tendencies were seen. ROC analysis based on death or survival for PNI yielded an AUC of 0.79; the corresponding sensitivity (%) and specificity (%) were 80% and 70%, respectively, and the most suitable reference value for PNI was 42.35, which is close to the consensus value of PNI 40 [16]. Considering the favorable results in ROC analyses for PNI, PNI may be a useful prognostic factor even in patients with CLD.

It must be stated that this study has several limitations. First, causality can be neither confirmed nor negated due to the observational study design. Second, this study was conducted with a Japanese population at a single institution and was retrospective by its nature. We have not studied non-Japanese ethnic groups. Additionally, in terms of survival analysis, various interventions for background CLD were given during the course of the follow-up period, which may have affected the prognosis and introduced bias. Despite these study limitations, the results of the current study indicate that PNI correlates well with ALBI score, and a lower PNI value may be a prognostic factor in patients with CLD.

## 5. Conclusions

PNI may be a useful tool for assessing liver function for CLD patients. A PNI score of <40 is helpful for the prediction of prognosis in patients with CLD.

## Figures and Tables

**Table 1:** Baseline features (*n* = 319).

	Number or Median (IQR)
Age (years)	71.0 (63.0, 78.0)
Gender, male/female	183/136
Etiology (Alcohol/HBV/HCV/MASLD/Others)	(39/52/73/112/43)
Cirrhosis, yes/no	256/63
BMI (kg/m^2^)	22.9 (20.8, 25.6)
Hb (g/dL)	12.8 (11.5, 14.1)
Serum albumin (g/dL)	3.8 (3.3, 4.1)
CRP (mg/dL)	0.18 (0.06, 0.69)
PT (%)	86 (74.0, 97.8)
Total lymphocyte count (/μL)	1259 (861, 1688)
ALT (IU/L)	27 (18.0, 55.0)
White blood cells (/μL)	5400 (4150, 6510)
Platelet count (×10^4^/mm^3^)	15.5 (11.1, 22.0)
eGFR (mL/min/1.73 m^2^)	65 (51.0, 80.0)
PNI	44.2 (38.7, 48.8)
PNI < 40, yes/no	94/225
Presence of HCC, yes/no	153/166
Total bilirubin (mg/dL)	0.8 (0.5, 1.2)
ALBI score	−2.48 (−2.76, −2.05)
ALBI grade, 1/2/3	122/176/21
Modified ALBI grade, 1/2a/2b/3	122/79/97/21
Child–Pugh score	6 (5, 7)
Child–Pugh classification (cirrhosis), A/B/C	171/77/8

IQR, interquartile range; HBV, hepatitis B virus; HCV, hepatitis C virus; MASLD, metabolic-dysfunction-associated steatotic liver disease; BMI, body mass index; Hb, hemoglobin; CRP, C-reactive protein; PT, prothrombin time; ALT, alanine aminotransferase; eGFR, estimated glomerular filtration rate; PNI, Prognostic Nutritional Index; HCC, hepatocellular carcinoma; ALBI, albumin–bilirubin.

**Table 2:** Univariate analysis.

	PNI ≥ 40 (*n* = 225)	PNI < 40 (*n* = 94)	*p* Value
Age (year)	70.0 (61.0, 78.0)	73.0 (68.0, 79.0)	0.0107
Gender (male/female)	122/103	61/33	0.0771
BMI (kg/m^2^)	22.81 (20.74, 25.54)	23.34 (20.92, 25.88)	0.3777
Hb (g/dL)	13.3 (12.2, 14.35)	11.5 (9.775, 12.6)	<0.0001
White blood cells (μL)	5480 (4345, 6495)	4960 (3797.5, 6655)	0.1399
CRP (mg/dL)	0.11 (0.04, 0.32)	0.775 (0.2275, 2.3025)	<0.0001
PT (%)	88 (78, 100)	79 (63, 91)	<0.0001
Platelet count (×10^4^/mm^3^)	160.0 (122.0, 228.5)	130.0 (86.5, 188.0)	0.0002
eGFR (mL/min/1.73 m^2^)	68.0 (55.0, 80.0)	54.0 (38.5, 73.0)	<0.0001
Presence of HCC, yes/no	97/128	56/38	0.0072
ALBI score	−2.62 (−2.87, −2.43)	−1.725 (−2.035, −1.4625)	<0.0001

Data are presented as the number or median value (interquartile range). BMI, body mass index; Hb, hemoglobin; CRP, C-reactive protein; PT, prothrombin time; eGFR, estimated glomerular filtration rate; PNI, Prognostic Nutritional Index; HCC, hepatocellular carcinoma; ALBI, albumin–bilirubin.

**Table 3:** Multivariate analysis.

Covariates	Multivariate Analysis		
	OR	95% CI	*p* Value
Age (per one year)	0.9932	0.9440–1.0451	0.7939
Gender (female)	2.8728	0.9822–8.4024	0.0550
BMI (per 1 kg/m^2^)	0.9961	0.8797–1.1278	0.9503
Hb (per 1 g/dL)	0.5977	0.4332–0.8248	0.0017
White blood cell (μL)	1.0000	0.9998–1.0002	0.8573
CRP (per 1 mg/dL)	1.2443	0.9528–1.6250	0.1085
PT (per 1%)	1.0133	0.9830–1.0445	0.3935
Platelet count (per 1 × 10^4^/mm^3^)	0.9958	0.9890–1.0027	0.2343
eGFR (per 1 mL/min/1.73 m^2^)	0.9778	0.9557–1.0003	0.0537
Presence of HCC	3.4805	1.0420–11.625	0.0426
ALBI score (per one)	903.74	133.34–6125.4	<0.0001

OR, odds ratio; CI, confidence interval; BMI, body mass index; Hb, hemoglobin; CRP, C-reactive protein; PT, prothrombin time; eGFR, estimated glomerular filtration rate; HCC, hepatocellular carcinoma; ALBI, albumin–bilirubin.

**Table 4:** Receiver operating characteristic curve analysis of independent parameters for PNI < 40.

PNI < 40	AUC	Sensitivity (%)	Specificity (%)	Cutoff Point
Hb	0.76	71.3	68.9	12.4
ALBI score	0.96	95.7	84.0	−2.34

PNI, Prognostic Nutritional Index; Hb, hemoglobin; ALBI, albumin–bilirubin; AUC, area under the receiver operating characteristic curve.

## Data Availability

The data presented in this study are not publicly available due to privacy or ethical restrictions.

## Abbreviations

CLD	chronic liver disease
PEM	protein–energy malnutrition
OMPU	Osaka Medical and Pharmaceutical University
IQR	interquartile range
PNI	Prognostic Nutrition Index
ALBI	albumin–bilirubin
mALBI	modified ALBI
ROC	receiver operating characteristic
OS	overall survival
HCC	hepatocellular carcinoma
AUC	area under the ROC curve
HBV	hepatitis B virus
HCV	hepatitis C virus
MASLD	metabolic-dysfunction-associated steatotic liver disease
SGA	subjective global assessment
